# Mammalian Ddi2 is a shuttling factor containing a retroviral protease domain that influences binding of ubiquitylated proteins and proteasomal degradation

**DOI:** 10.1016/j.jbc.2022.101875

**Published:** 2022-03-28

**Authors:** Galen Andrew Collins, Zhe Sha, Chueh-Ling Kuo, Beyza Erbil, Alfred L. Goldberg

**Affiliations:** Department of Cell Biology, Blavatnik Institute, Harvard Medical School, Boston, Massachusetts, USA

**Keywords:** Ddi2, 26S proteasome, unfolded protein response, nelfinavir, aspartic protease, shuttling factor, HIV protease inhibitor, protein degradation, ubiquitin-like domain, polyubiquitin chain, BiP, binding immunoglobulin protein, BSA, bovine serum albumin, DMSO, dimethyl sulfoxide, DUb, deubiquitylating enzyme, ER, endoplasmic reticulum, ERAD, endoplasmic reticulum–associated degradation, FBS, fetal bovine serum, GST, glutathione-*S*-transferase, HA, hemagglutinin, HEK293, human embryonic kidney 293 cell line, Hsp, heat shock protein, Ig, immunoglobulin, NeD-sfGFP, super-folder GFP with an N-end degron, NTA, nitrilotriacetic acid, TCA, trichloroacetic acid, TCEP, Tris(2-carboxyetheyl)phosphine, Ub, ubiquitin, UBA, Ub-associated domain, UBL, Ub-like domain, UBQLN1, ubiquillin-1, UIM, Ub-interacting motif, UPR, unfolded protein response

## Abstract

Although several proteasome subunits have been shown to bind ubiquitin (Ub) chains, many ubiquitylated substrates also associate with 26S proteasomes *via* “shuttling factors.” Unlike the well-studied yeast shuttling factors Rad23 and Dsk2, vertebrate homologs Ddi2 and Ddi1 lack a Ub-associated domain; therefore, it is unclear how they bind Ub. Here, we show that deletion of Ddi2 leads to the accumulation of Ub conjugates with K11/K48 branched chains. We found using affinity copurifications that Ddi2 binds Ub conjugates through its Ub-like domain, which is also required for Ddi2 binding to proteasomes. Furthermore, in cell extracts, adding Ub conjugates increased the amount of Ddi2 associated with proteasomes, and adding Ddi2 increased the binding of Ub conjugates to purified proteasomes. In addition, Ddi2 also contains a retroviral protease domain with undefined cellular roles. We show that blocking the endoprotease activity of Ddi2 either genetically or with the HIV protease inhibitor nelfinavir increased its binding to Ub conjugates but decreased its binding to proteasomes and reduced subsequent protein degradation by proteasomes leading to further accumulation of Ub conjugates. Finally, nelfinavir treatment required Ddi2 to induce the unfolded protein response. Thus, Ddi2 appears to function as a shuttling factor in endoplasmic reticulum–associated protein degradation and delivers K11/K48-ubiquitylated proteins to the proteasome. We conclude that the protease activity of Ddi2 influences this shuttling factor activity, promotes protein turnover, and helps prevent endoplasmic reticulum stress, which may explain nelfinavir’s ability to enhance cell killing by proteasome inhibitors.

Although 26S proteasomes can directly bind to ubiquitylated proteins with high affinity and degrade them efficiently, many ubiquitylated substrates bind to proteasomes through protein intermediates (1, 2). In yeast, the shuttling factors Rad23, Dsk2, and Ddi1 bind ubiquitin (Ub) chains through their C-terminal Ub-associated (UBA) domains (3, 4, 5, 6, 7) and associate with the proteasome's 19S regulatory complex through their N-terminal Ub-like (UBL) domains (8, 9, 10). However, the need for these additional binding factors and their specific roles are still unclear. These shuttling factors may function as extrinsic Ub receptors (1), creating additional Ub-binding platforms on the proteasome, or they may serve as shuttling factors, which bind Ub conjugates and help deliver them to the 26S proteasome (11, 12, 13) while protecting their Ub chains from destruction by cytosolic deubiquitylating enzymes (DUbs) (14). We recently discovered that the UBL domains on these proteins also activate the proteasomes’ ATPase and peptidase activities (15, 16), which implies that these factors have an important role in promoting proteolysis beyond merely substrate delivery.

While Rad23, Dsk2, and their mammalian homologs have been studied frequently, much less is known about the functions of the Ddi1 family. Yeast Rad23 and Dsk2 bind Ub chains with high affinity and are found in association with most of the ubiquitylated proteins on the 26S proteasome (17). By contrast, Ddi1 seems to bind very few Ub chains under similar conditions (17). Similarly, while Rad23 and Dsk2 are clearly important in endoplasmic reticulum (ER)–associated degradation (ERAD) of misfolded proteins, Ddi1 was not found to serve a similar role in this process (12). Thus, its possible contributions to protein degradation remain unclear.

Furthermore, the Ddi1 family of proteins has several features, which seem inconsistent with a role as a Ub receptor. Although in *Saccharomyces cerevisiae*, Ddi1 contains the typical UBL and UBA domains, Ddi1 family members in many other organisms have not preserved this domain architecture. In mammals, there are two Ddi1 family members—Ddi2, which is expressed in most cells, and Ddi1, whose expression is limited. Both lack a C-terminal UBA domain, and therefore, if they function as shuttling factors, they must have another mechanism for Ub recognition. Nowicka *et al.* (18) reported that the yeast Ddi1 UBL domain has an unusual though weak affinity for di-Ub chains, and because Ddi2 forms dimers (19), they proposed that one UBL domain binds proteasomes, whereas the other UBL domain could bind Ub chains. In mammals, Ddi2 has a noncanonical Ub-interacting motif (UIM) at its C terminus, which shows a low affinity for Ub but has been proposed to enable Ddi2 to bind Ub chains (20). Despite the presence of these two weak Ub-binding mechanisms, it remains unclear how vertebrate Ddi2 and Ddi1 can simultaneously bind to proteasomes and Ub conjugates with sufficient affinity to serve as shuttling factors and to promote conjugate degradation.

If proteins like Rad23 or Ddi2 are shuttling factors rather than simply extrinsic Ub receptors, they ought to have several key properties, in addition to simultaneously binding ubiquitylated proteins and proteasomes. The term shuttling factor implies a delivery process from one site (*e.g.*, the ER or the unfoldase Cdc48/p97) to proteasomes (12, 13). Accordingly, the binding of known shuttling factors to proteasomes in cell lysates is markedly stimulated by the presence of Ub conjugates (21), even though as purified proteins shuttling factors of readily bind isolated proteasomes (10, 14, 22). Furthermore, as the ubiquitylated substrate is processed by the proteasome, shuttling factors like Rad23B dissociate from the proteasome (21). These findings support a role for shuttling factors in bringing substrates to proteasomes but indicate some unknown step regulating their binding to proteasomes. Furthermore, Rad23A and Rad23B can activate proteasomes when bound to the 26S particles (15, 16). We found that Ddi2 similarly activates proteasomes (15), but it is unclear if Ddi2 possesses the other characteristic features of a shuttling factor.

The Ddi1 family possesses another very peculiar feature that has recently stimulated appreciable interest. Unlike Rad23B or ubiquilins (UBQLNs; the mammalian Dsk2 homologs), the Ddi1 protein family has an aspartyl protease domain that closely resembles the active sites of the acidic proteases from retroviruses, especially HIV-1 (23). It is unexpected and intriguing that a protein that supposedly delivers ubiquitylated proteins to proteasomes should also have protease activity. Thus far, only two physiological substrates of the Ddi2 protease have been identified: the transcription factors Nrf1/NFEL2L1/TCF11 and Nrf3/NFE2L3 (24, 25, 26). In response to proteasome inhibition, these ER-associated proteins are cleaved by Ddi2, and their C-terminal fragments enter the nucleus and catalyze the transcription of proteasome genes. Similarly, in *Caenorhabditis elegans*, proteasome inhibition leads to the cleavage of the homologous transcription factor Skn-1a by Ddi-1, which also promotes the production of new proteasomes (27). Recently, yeast Ddi1 and mammalian Ddi2 were demonstrated to be Ub-dependent endoproteases (28, 29), supporting our earlier prediction based on the observation that Ddi2 cleaves Nrf1 after its ubiquitylation (25). This unusual Ub dependence probably explains why earlier screens for Ddi2 substrates have been unsuccessful. However, there must exist other important endogenous targets of this protease. For example, yeasts lack an Nrf1 homolog and have a distinct mechanism to activate proteasome gene expression, which does not involve Ddi1 (30). Thus, the protease activity of Ddi1 must have other substrates in yeast.

It is unclear whether this potential shuttling factor role of Ddi2 is linked to its proteolytic activity or even if this activity somehow contributes to overall protein degradation by the Ub–proteasome pathway. Because the Ddi2 protease resembles retroviral proteases, an inhibitor of the HIV-1 protease, nelfinavir, has been tested and found to inhibit yeast Ddi1 (28) and human Ddi2 (31). This agent was also found to enhance cell killing by proteasome inhibitors (31, 32). Consequently, nelfinavir is being studied in clinical trials in combination with proteasome inhibitors to treat multiple myeloma (33). However, several potential cellular targets of nelfinavir other than Ddi2 were proposed, although none have been confirmed (34).

The present studies were undertaken to determine if Ddi2 can indeed function as a shuttling factor like Rad23B. We first investigated if Ddi2 binds ubiquitylated proteins and determined which domain might contribute to the binding and tested in cell extracts if Ddi2 actually promotes the binding of Ub conjugates to proteasomes. We were surprised to find that Ddi2 deletion causes an accumulation in cells of Ub conjugates that migrate unusually slowly upon SDS-PAGE. The apparent size of ubiquitylated proteins increased after Ddi2 deletion from a range of 130 to 200 kDa to much larger than 240 kDa. Similar slowly migrating Ub conjugates have been shown to be degraded very rapidly by proteasomes (35) and have been found on proteins degraded by the ERAD pathway (36), in which shuttling factors appear to take ubiquitylated proteins from p97 and deliver them to proteasomes (13). Their slow migration was shown to be due to the formation of branched Ub chains with linkages to K11 and K48 residues on the same Ub. Therefore, we studied whether deleting Ddi2 leads to the formation of branched chains and also tested if inactivation or inhibition of the proteolytic site of Ddi2 influences the binding of Ddi2 to Ub chains and proteasomes. The availability of the inhibitor, nelfinavir, enabled us also to determine whether Ddi2 influences overall rates of protein breakdown in the cell and degradation by the Ub–proteasome pathway. Finally, because nelfinavir induces the unfolded protein response (UPR) (37), we tested whether nelfinavir causes ER stress through an inhibition of the proteolytic activity of Ddi2.

## Results

### Ddi2 binds preferentially to slowly migrating Ub conjugates

**Figure 1:**
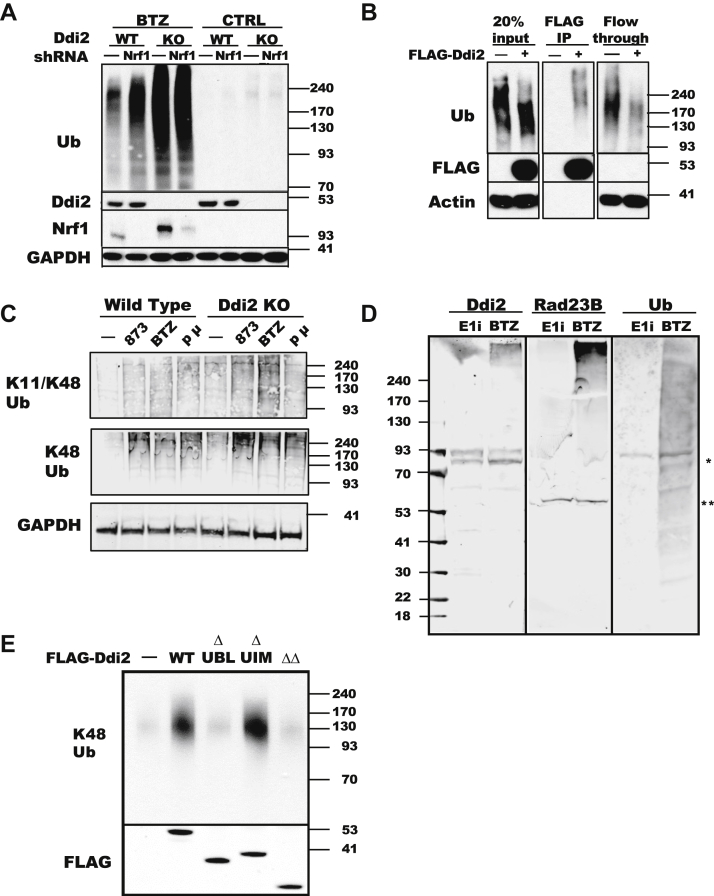
**Ddi2 binds large ubiquitylated proteins *via* its UBL domain.***A*, deletion of Ddi2 leads to an accumulation of Ub conjugates, especially large (slowly migrating) conjugates. Immunoblots of WT or Ddi2 knockout HAP1 cells treated with 100 nM bortezomib (BTZ) for 16 h in cells stably expressing GFP (−) or shRNA to Nrf1. Molecular weights here, and throughout, are shown as kilodalton based on the apparent migration size of BLUEstain Protein Ladder (Gold Biotechnology, Inc). *B*, Ddi2 preferentially binds large Ub conjugates. About 20% input, FLAG-IP, and unbound fractions from HEK293A cells transfected with FLAG-Ddi2 (+) or with an empty vector (–). *C*, knockout of Ddi2 leads to increased K11/K48 branched chains. HAP1 cells were treated with 5 μM of the p97 inhibitor NMS-873 (873), 1 μM of the proteasome inhibitor BTZ, or 10 μM of the Hsp90 inhibitor pifithrin μ (p μ) for 5 h. A representative image of three blot separate experiments is shown. *D*, Ddi2, like Rad23B, binds preferentially to large Ub conjugates. Lysates from HAP1 Ddi2 knockout cells treated with 100 nM BTZ for 16 h (to cause accumulation of Ub conjugates) or 5 μM TAK-243 (E1i) for 2 h (to eliminate ubiquitylated proteins) were run on SDS-PAGE gels. Membranes were probed with recombinant Ddi2 or Rad23B protein and blotted with antibodies against Rad23B, Ddi2, or Ub (P4D1). *Asterisks* denote likely crossreaction with endogenous Ddi2 (∗) or Rad23B (∗∗). A representative of two independent experiments is shown. *E*, the Ddi2 UBL domain is necessary for binding Ub conjugates. Immunoblots of FLAG affinity pulldown from lysates of HEK293A cells expressing full-length FLAG-Ddi2 (WT), ΔUBL truncation, ΔUIM truncation, or the double deletion of UBL and UIM (ΔΔ).HEK293A, human embryonic kidney 293A cell line; IP, immunoprecipitation; Ub, ubiquitin; UBL, Ub-like domain; UIM, Ub-interacting motif.

While studying the proteolytic activation of Nrf1 by Ddi2 (25), we noticed that the deletion of Ddi2 in HAP1 cells led to an accumulation of Ub conjugates that appeared unusually large by SDS-PAGE immunoblots (Fig. 1*A*). These Ub conjugates migrated more slowly (*i.e.*, they appeared much larger than 240 kDa) than the conjugates that accumulate when cells are treated with the proteasome inhibitor, bortezomib, which appeared to be between 130 and 200 kDa (Fig. 1*A*), as was also reported by Dirac-Svejstrup *et al.* (29) in MRC5VA Ddi2 deletion cells. To determine if the lack of the transcription factor Nrf1, one of the two confirmed targets of Ddi2 and an important regulator of proteasome gene expression, caused the buildup of these ubiquitylated proteins, we used shRNA to knockdown Nrf1 in both WT and Ddi2-deleted HAP1 cell lines. Knockdown of Nrf1 did not lead to a similar accumulation of slowly migrating conjugates in the WT cells and did not affect the buildup of large conjugates in the Ddi2 deletion strains (Fig. 1*A*). In addition, deletion of Ddi2 prevents the cleavage of Nrf1, as previously observed (24, 27). Thus, Ddi2 either inhibits the formation or promotes the degradation of these large conjugates by a mechanism that does not involve decreased Nrf1 activity or a reduction in proteasome amount or activities.

If Ddi2 is a shuttling factor bringing ubiquitylated substrates to the 26S proteasomes, then Ddi2 should bind strongly ubiquitylated proteins and in light of Figure 1*A* perhaps bind large conjugates preferentially. Therefore, we expressed FLAG-Ddi2 in human embryonic kidney 293A (HEK293A) cells and analyzed by immunoblot, which proteins coprecipitated with Ddi2. After the expression of FLAG-Ddi2, the sizes of Ub conjugates appeared to be smaller than in the untransfected cells (Fig. 1*B*). However, the Ub conjugates that coprecipitated with FLAG-Ddi2 migrated slowly, even more slowly than the conjugates in the untransfected cells (input) and in the flow-through fractions from control-transfected or Ddi2-transfected cells (Fig. 1*B*).

The slowly migrating Ub chains that accumulate upon Ddi2 deletion may arise through three possible mechanisms. The substrates for Ddi2 could be abnormally large proteins, (*i.e.*, on the average, more than 100 kDa larger than ubiquitylated proteins that accumulate upon proteasome inhibition (Fig. 1*A*)). Alternatively, the Ub chains in these conjugates could be extraordinarily long, bearing on the average 11 additional Ub molecules. Possibly, Ddi2 deletion might increase the length of Ub chains by not restricting chain elongation by Ub ligases, as was reported to occur with Rad23 (22, 38). A more likely alternative in our view is that these ubiquitylated proteins migrate slowly because their Ub chains are extensively branched. Proteins bearing branched Ub chains tend to be degraded particularly rapidly (35), and branched Ub chains have been observed on ERAD substrates (36). Therefore, we tested if the deletion of Ddi2 might cause a buildup of a particular type of branched Ub chains formed by modification of both lysines 11 and 48 on a single Ub (K11/K48) by using a bispecific antibody that recognizes branched K11/K48 chains (39). In WT cells, the levels of K11/K48 branched chains are low but increase with inhibition of p97 (NMS-873), of proteasomes (bortezomib) or of heat shock protein 90 (Hsp90) (pifithrin μ) (Fig. 1*C*). When Ddi2 was knocked out, the levels of K11/K48 branched chains markedly increase to levels nearly as high as we obtained with p97 or proteasome inhibition (Fig. 1*C*). Thus, the slowly migrating ubiquitylated proteins contain some branched K11/K48 Ub chains, and Ddi2 has a previously unrecognized role in restricting the abundance of these chains.

To determine if the preferential binding of Ddi2 to slowly migrating ubiquitylated proteins is unique to Ddi2, or if it is a feature of other shuttling factors, we used “far-Western” immunoblots to monitor the binding of Ddi2 and Rad23B to ubiquitylated proteins. HAP1 Ddi2 knockout cells were treated with 100 nM bortezomib overnight to provide an abundant source of these unusually large Ub conjugates. We also included as controls, lysates from cells treated with 1 μM of the E1 inhibitor TAK-243 for 1 h to obtain lysates without significant levels of ubiquitylated proteins. After the membranes were probed with either purified Ddi2 or Rad23B, we used antibodies to detect whether Ddi2 and Rad23B bound preferentially to large conjugates. Both Ddi2 and Rad23B bound to proteins migrating at the very top of the gel (Fig. 1*D*). Because the E1 inhibitor prevented this binding of Ddi2 and Rad23B, these factors must be binding to ubiquitylated proteins. Moreover, the Ub conjugates bound by Ddi2 and Rad23B appear to be much larger than the average ubiquitylated proteins detected in the cells by the pan-Ub Western blot (Fig. 1*D*).

### Ddi2 binds Ub conjugates through its UBL domain

The well-characterized shuttling factors in yeast are UBL–UBA proteins that bind Ub conjugates through their UBA domain and bind to proteasomes by their UBL domain. However, since Ddi2 in humans and other vertebrates lacks a UBA domain, two models have been proposed to explain how Ddi2 might bind Ub chains. Nowicka *et al.* (18) reported that the yeast Ddi1 can bind to Ub through the UBL domain at its N terminus, whereas Sivá *et al.* (20) suggested that a potential UIM is found at the C terminus of Ddi2 in some mammals, including humans. Both studies demonstrated weak binding (*K*_*a*_ ∼ 10–100 μM) of Ddi2 to mono-Ub or di-Ub molecules, rather than to the large conjugates to which Ddi2 preferentially binds (Fig. 1*D*). Therefore, we tested if the UBL, UIM, or both domains were necessary for Ddi2 to bind ubiquitylated proteins by expressing in cells FLAG-Ddi2 with each of these domains deleted (ΔUBL, ΔUIM) as well as deleting both (ΔΔ). Even if the UIM domain was deleted from Ddi2, Ub conjugates could be precipitated with FLAG-Ddi2 as with the WT Ddi2 (Fig. 1*E*). In contrast, deletion of the UBL domain eliminated the ability of Ddi2 to precipitate Ub, and no further change was evident if the UIM domain was deleted together with the UBL domain (Fig. 1*E*). Thus, Ddi2 binds through its UBL domain to slowly migrating Ub conjugates, which accumulate if Ddi2 is deleted.

### In cells, Ub conjugates promote the binding of Ddi2 to proteasomes

**Figure 2:**
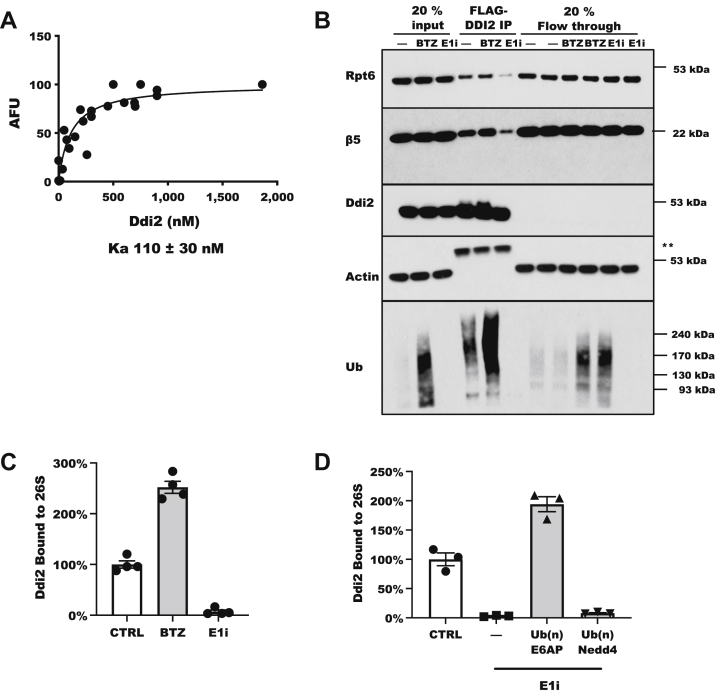
**In cells, Ub conjugates promote the binding of Ddi2 to proteasomes.***A*, purified His_6_-Ddi2 binds to purified 26S proteasomes with high affinity. The amount of UBL affinity purified 26S proteasomes bound to varying concentrations of His_6_-Ddi2 as determined by suc-Leu-Leu-Val-Tyr-amc hydrolysis reported as arbitrary fluorescent units (AFUs) with individual data points from three independent experiments combined. *B*, In cell lysates, ubiquitylated proteins increase the binding of FLAG-Ddi2 to 26S proteasomes (indicated by β5 and Rpt6 subunits). Immunoblots of pulldown of FLAG-Ddi2 in HEK293A cells treated for 4 h with 10 μM bortezomib (BTZ) to cause a buildup of Ub conjugates on proteasomes or 10 μM TAK-243 (E1i) to eliminate Ub conjugates. (∗∗ denotes IgG heavy chain from the immunoprecipitation). *C*, the binding of Ddi2 to 26S proteasomes in cell lysates depends on ubiquitylated proteins. Quantification of immunoblots for Ddi2 on Dss1-FLAG–purified 26S proteasomes from HEK293F DSS1-FLAG cells after 30 min of 1 μM BTZ or 1 h of 1 μM E1i. Rpt5 content is used as a measure of 26S proteasomes, and the ratio of Ddi2/Rpt5 is expressed as the percent of untreated control cells (n = 4; mean ± SEM). *D*, K48-linked, but not K63-linked, Ub conjugates added to cell lysates increase the binding of Ddi2 to 26S proteasomes. About 100 nM ubiquitylated GST-E6AP (K48 chains) or ubiquitylated GST-Nedd4 (K63 chains) were added to lysates of HEK293F cells treated with 1 μM E1i for 1 h and incubated at 4 °C for 1 h. Quantification of immunoblots for Ddi2 on Dss1-FLAG-purified 26S proteasomes is shown. The Ddi2/Rpt5 ratio is expressed as the percent of untreated control cells (n = 3; mean ± SEM). HEK293, human embryonic kidney 293 cell line; IgG, immunoglobulin G; Ub, ubiquitin; UBL, Ub-like domain.

To measure the binding of Ddi2 to 26S proteasomes, we used purified His_6_-Ddi2 and 26S proteasomes isolated by the UBL affinity method, which yields proteasomes without significant bound Ub conjugates (40). As we reported previously (15), Ddi2 binds to 26S proteasomes with high affinity (*K*_*a*_ ∼ 110 nM) (Fig. 2*A*). Previously, we discovered that even though purified Rad23B binds similarly to proteasomes with high affinity (15, 41), in cell lysates, the strong association of Rad23B with proteasomes required Ub conjugates to be present (21). To determine if Ub conjugates are also needed for the binding of Ddi2 to proteasomes in cell lysates, we transfected HEK293A cells with FLAG-Ddi2 and measured by immunoblot the amounts of Rpt6 (a representative 19S proteasome regulatory subunit) and β5 (a representative 20S subunit) that associated with FLAG-Ddi2. Both the 19S and 20S subunits associated with Ddi2 (Fig. 2*B*) and their levels on Ddi2 increased when we increased Ub conjugate levels by treating cells with bortezomib. Conversely, when we treated the cells with the E1 inhibitor TAK-243 to greatly reduce ubiquitylated proteins, much fewer proteasomes were bound to Ddi2 (Fig. 2*B*).

Similarly, using FLAG-tagged Rpn11 proteasomes, we measured the binding of endogenous Ddi2 by immunoblot to FLAG 26S proteasomes by affinity pulldown. If the lysates were from cells treated with bortezomib, which was added to cause ubiquitylated proteins to accumulate, then the amount of Ddi2 on the 26S proteasomes nearly doubled (Fig. 2*C*). Conversely, if the lysates were from cells treated with the E1 inhibitor TAK-243, which greatly reduces levels of Ub conjugates, then Ddi2 was undetectable on the 26S proteasomes (Fig. 2*C*). Moreover, if we added back a defined Ub conjugate, Ub(n)E6AP, to the lysates treated with the E1 inhibitor, then we restored the binding of endogenous Ddi2 to FLAG-affinity-bound 26S proteasomes (Fig. 2*D*). By contrast, the addition of ubiquitylated Nedd4, which is principally composed of K63-linked ubiquitin chains (42), did not increase the binding of Ddi2 to 26S proteasomes (Fig. 2*D*). Thus, although purified Ddi2 can bind to isolated 26S proteasomes, in cell lysates, their association depends on the presence of ubiquitylated proteins, which are presumably substrates that Ddi2 delivers to the proteasome for degradation.

### Ddi2 increases Ub conjugate binding to proteasomes and dissociates from proteasomes as the conjugates are degraded

**Figure 3:**
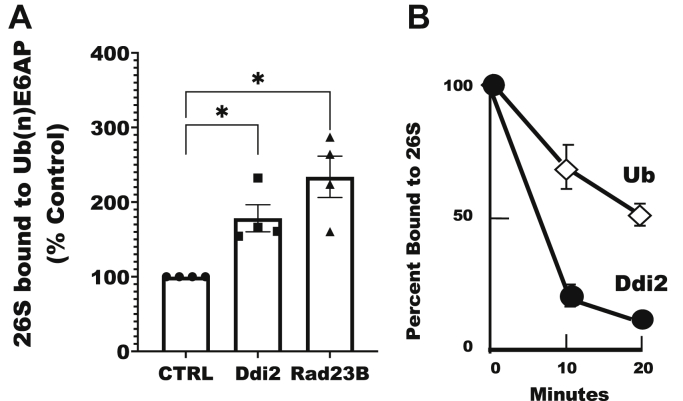
**Ddi2 increases binding of ubiquitylated proteins to proteasomes and dissociates from proteasomes as Ub conjugates are hydrolyzed.***A*, purified Ddi2, like purified Rad23B, increases the binding of purified 26S proteasomes to ubiquitylated E6AP immobilized on GSH-sepharose. 26S proteasomes bound to ubiquitylated E6AP, either without any shuttling factor added or with 500 nM Ddi2 or Rad23B present, were detected by measuring the hydrolysis of suc-Leu-Leu-Val-Tyr-amc (n = 4; individual points with mean as a horizontal bar; ∗*p* < 0.05). *B*, as proteasomes degraded the bound Ub conjugates, Ddi2 dissociated from the proteasomes. Dss1-FLAG–purified 26S proteasomes were isolated at 4 °C from HEK293F cells with bound conjugates and then were incubated at 37 °C before washing. Quantification of immunoblots for bound Ub (*open diamonds*) or Ddi2 (*filled circles*). Levels of proteins remaining on proteasomes were normalized to the level of the proteasome subunit Rpt5, and the amount of protein at *t* = 0 was set to 100% (n = 5; mean ± SEM). HEK293F, human embryonic kidney 293F cell line; Ub, ubiquitin.

Because Ddi2 uses its UBL domain to bind both to Ub conjugates (Fig. 1*E*) and to proteasomes (43), it was important to test if Ddi2 actually can bind both simultaneously as is necessary for it to function as a shuttling factor. Nathan *et al.* (41) showed that Rad23B and Rad23A could stimulate the binding of 26S proteasomes to ubiquitylated E6AP. Similarly, addition of purified Ddi2 increased the binding of proteasomes to ubiquitylated E6AP (Fig. 3*A*). The function of a shuttling factor, such as Rad23B, also requires its dissociation during conjugate hydrolysis (21). We therefore monitored the fate of Ddi2 bound to Ub conjugates on Dss1-FLAG-tagged proteasomes. The 26S proteasomes with bound Ub conjugates and Ddi2 were captured by anti-FLAG resin at 4 °C, and then the resin was shifted to 37 °C. As the proteasomes deubiquitylated or degraded the substrates, Ddi2 dissociated from the proteasomes (Fig. 3*B*). Thus, Ddi2 behaves much like the known shuttling factor Rad23B, being able to promote the binding of ubiquitylated proteins to 26S proteasomes and then dissociating as the Ub conjugates are hydrolyzed.

### Ddi2 is a retroviral protease inhibited by the HIV protease inhibitor nelfinavir

**Figure 4:**
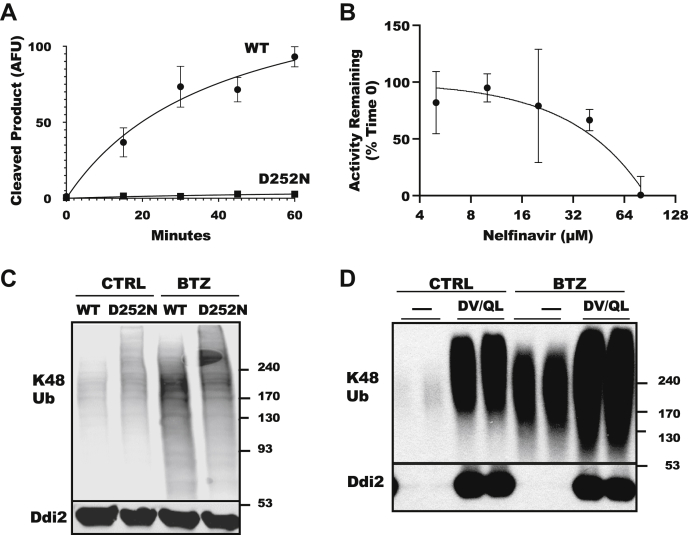
**Ddi2 is a protease active against a ubiquitylated protein, and its inhibition results in an accumulation of large Ub conjugates.***A*, purified WT Ddi2 is an endoprotease that cuts ubiquitylated NeD-sfGFP (62). His6-Ddi2 (WT) and the active site mutant His6-Ddi2 D252N (D252N) were incubated with 100 nM labeled NeD-sfGFP. Cleavage of the NeD-sfGFP by Ddi2 releases a short fluorescent product that can be separated from full-length NeD-sfGFP by SDS-PAGE and quantified by an Odyssey CLx imager (n = 3; mean ± SEM). *B*, the HIV retroviral protease inhibitor nelfinavir inhibits Ddi2. Cleavage of ubiquitylated NeD-sfGFP by His_6_-Ddi2 with nelfinavir is shown as percent of the activity without the drug (n = 3; mean ± SEM). *C*, cells with proteolytically inactive Ddi2 D252N accumulated slowly migrating Ub conjugates. Triton X-100 soluble lysates of HCT116 Ddi2 WT or Ddi2 D252N cells were treated with 100 nM bortezomib (BTZ) or DMSO (CTRL) for 16 h. *D*, inactive Ddi2 leads to the accumulation of large Ub conjugates. Lysates from HEK293A cells transfected with the double mutant Ddi2 D252V Q256L (DQ/VL) for 2 days prior to treatment with 100 nM BTZ or DMSO (CTRL) for 16 h. DMSO, dimethyl sulfoxide; HEK293A, human embryonic kidney 293A cell line; NeD-sfGFP, super-folder GFP with an N-end degron.

Ddi2 is unusual among shuttling factors in that it has an acidic protease domain. In *C. elegans*, the homolog of Nrf1, Skn-1a, is cleaved by Ddi-1 (27), and in mammals, Ddi2 is necessary for the proteolytic processing of Nrf1 in cells (24, 25), which occurs after Nrf1 has been polyubiquitylated (29). Yip *et al.* (28) showed that yeast Ddi1 can cleave ubiquitylated super-folder GFP with an N-end degron (NeD-sfGFP), and that the HIV protease inhibitor, nelfinavir, can inhibit this cleavage. We tested if human Ddi2 could also cut this fluorescent protein. WT Ddi2 did indeed hydrolyze this substrate, but Ddi2 with the active site aspartate 252 mutated to an asparagine (Ddi2 D252N) did not digest this substrate (Fig. 4*A*). Furthermore, the addition of nelfinavir inhibited its cleavage (Fig. 4*B*). Thus, human Ddi2 is a Ub-dependent endoprotease sensitive to nelfinavir. The EC_50_ value for nelfinavir obtained from these experiments, ∼40 μM, resembles that observed with the yeast Ddi1 (28). However, the EC_50_ appears greater than the levels needed to inhibit Nrf1 cleavage in cells (31, 32), perhaps because the concentration of the substrate NeD-sfGFP was high (100 nM) and equal to the monomeric concentration of Ddi2 in the reaction.

### Loss of the Ddi2 protease activity causes Ub conjugate accumulation

To test if the Ddi2 protease affects protein degradation rates in cells, we first studied HCT116 cells with a CRISPR knock-in of the Ddi2 D252N mutation, kindly provided by Professor Shigeo Murata (24). Like the Ddi2 knockout cells, this point mutation caused the accumulation of slowly migrating Ub conjugates (Fig. 4*C*). This effect of the Ddi2 point mutation is quite different from the consequences of inhibiting the proteasome in WT cells with bortezomib, which increased the levels of ubiquitylated proteins, generally and not just slowly migrating conjugates. However, treating the Ddi2 D252N mutant with bortezomib increased preferentially the levels of those slowly migrating Ub conjugates (Fig. 4*C*). Similarly, the overexpression of another protease-deficient mutant, Ddi2 D252V Q256L (DV/QL), in HEK293A cells also caused a buildup of Ub conjugates, especially ones that migrate very slowly (Fig. 4*D*).

**Figure 5:**
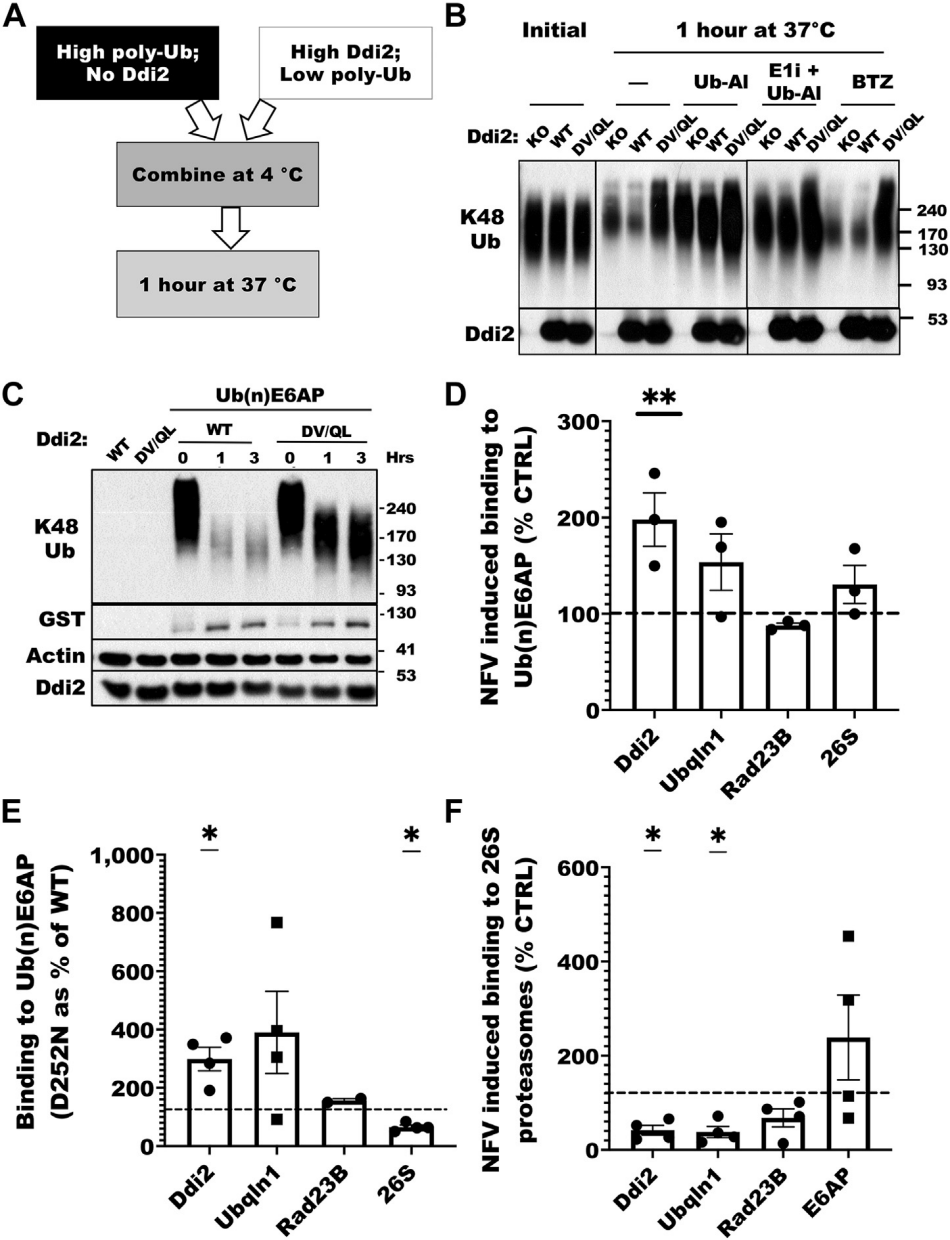
**Inhibiting the retroviral protease site of Ddi2 enhances the binding of Ddi2 to Ub conjugates but decreases binding to proteasomes.***A*, the design of the experiment in (*B*) to test the effects of the Ddi2 protease activity on its association with Ub conjugates. Lysates of HAP1 Ddi2 knockout cells were treated with 1 μM bortezomib (BTZ) for 4 h (to obtain a high content of Ub conjugates), but no Ddi2 was mixed with lysates of HAP1 Ddi2 knockout cells transfected with WT Ddi2 or the inactive mutant D252V Q256L Ddi2 (DV/QL) and treated with 10 μM of the E1 inhibitor TAK-243 (Eli) for 4 h (to obtain high levels of WT or inactive Ddi2 and no Ub conjugates). After these lysates were combined, the lysates were incubated at 37 °C for 1 h, and the changes in levels of the Ub conjugates were analyzed at different times. *B*, inactive Ddi2 protects Ub conjugates from deubiquitylation. Lysates containing ubiquitylated proteins but no Ddi2 were mixed with lysates containing high levels of Ddi2, but lacking Ub conjugates as described in (*A*). The mixed lysates (*left three lanes*) were incubated at 37 °C for 1 h with 1.6 μM Ub aldehyde (Ub-Al) to block DUb activity and 10 μM E1i to prevent further ubiquitylation, or 10 μM BTZ, which allows DUb function but blocks proteasomal degradation. *C*, inactive Ddi2 protects ubiquitylated E6AP from deubiquitylation. HAP1 Ddi2 knockout cells were transfected with WT or D252V Q256L Ddi2 and treated with 10 μM of the E1 inhibitor Eli for 4 h as in (*B*). Ubiquitylated GST-E6AP was added to the lysates and incubated at 37 °C for up to 3 h. *D*, inhibition of the proteolytic activity of Ddi2 with nelfinavir (10 μM, 30 min) increases the binding of Ddi2 and UBQLN1 but not Rad23B to ubiquitylated E6AP. Lysates of HEK293-HBTH-Rpn1 were incubated with ubiquitylated GST-E6AP on immobilized glutathione-Sepharose for 1 h at 4 °C, and immunoblots for different proteins were quantitated. *E*, mutation of the Ddi2 protease site increases the binding of Ddi2 to ubiquitylated E6AP. Lysates of WT Ddi2 and D252N mutant HCT116 cells were incubated as in (*D*) with ubiquitylated E6AP. Quantification of immunoblots of Ddi2 that bound to ubiquitylated GST-E6AP as the percent binding of proteins from mutant cells *versus* the WT. *F*, inhibition of the Ddi2 protease site with nelfinavir decreases the binding of Ddi2 to 26S proteasomes. HEK293-HBTH-Rpn11 cells were treated with 10 μM nelfinavir or with vehicle (methanol) for 30 min, and lysates were incubated with streptavidin resin to bind 26S proteasomes. The amount of Ddi2 present was then assayed. (n = 4; mean; ∗*p* < 0.05, ∗∗*p* < 0.01, ns). DUb, deubiquitylating enzyme; GST, glutathione-*S*-transferase; HEK293, human embryonic kidney 293 cell line; ns, not significant; Ub, ubiquitin.

To determine how the Ddi2 protease domain may be affecting the level of Ub conjugates, we mixed (Fig. 5*A*) lysates that had high levels of ubiquitylated proteins and no Ddi2 with lysates that had high levels of Ddi2 and very little or no Ub conjugates. To obtain lysates with high levels of ubiquitylated proteins, we treated HAP1 Ddi2 knockout cells with 10 μM bortezomib for 4 h. We prepared lysates with little or no Ub conjugates by treating HAP1 cells transfected with Ddi2 (WT or inactive D252V Q256L DV/QL) with the E1 inhibitor TAK-243. After these lysates were combined and incubated at 37 °C for an hour, we measured Ub conjugates by immunoblot. In the mixture with WT Ddi2 or no Ddi2, Ub conjugates, especially the slowly migrating conjugates, disappeared rapidly (Fig. 5*B*). However, in the mixes containing the DV/QL inactive Ddi2, the levels of slowly migrating Ub conjugates did not change. On the contrary, the DV/QL mutant seemed to protect against the cytosolic DUbs. Thus, the WT Ddi2, but not the inactive mutant, seems to promote the metabolism of the larger Ub conjugates.

To determine if DUbs caused the loss of these large conjugates, the broad-spectrum DUb inhibitor, Ub aldehyde, was added. This agent inhibited the disappearance of the Ub conjugates, especially in the lysate mixture containing the active Ddi2 (Fig. 5*B*). However, the migration of the remaining conjugates still appeared slower than in the initial mixtures. This effect was not because of further ubiquitylation or chain extension, since the slowly migrating species were still evident when the DUb inhibitor was added with an E1 inhibitor (Fig. 5*B*). To determine if proteasome degradation contributed to the loss of these Ub conjugates, bortezomib was included in the incubation. However, under those conditions, the mutant Ddi2 (DV/QL) still maintained the slow migration pattern of ubiquitylated proteins (Fig. 5*B*). Thus, mutating the active site of Ddi2 protease appears to protect the slowly migrating conjugates from cellular DUbs.

To verify these surprising findings, we incubated ubiquitylated glutathione-*S*-transferase (GST)-E6AP (Ub(n)E6AP) in lysates of Ddi2 knockout HAP1 cells transfected with either WT or the inactive DV/QL mutant and treated the cells with TAK-243 to eliminate endogenous Ub chains. Incubation of Ub(n)E6AP in the lysates containing WT Ddi2 at 37 °C led to extensive deubiquitylation (Fig. 5*C*). In contrast, in the Ddi2 mutant lysates, some deubiquitylation of E6AP occurred but much less than in the presence of WT Ddi2 (Fig. 5*C*). Thus, the Ddi2 mutant, unlike the WT, can protect ubiquitylated proteins from cellular DUbs.

### The protease activity of Ddi2 affects the binding of Ddi2 to Ub conjugates and proteasomes

One possible explanation of these findings is that inactivation of the proteolytic site of Ddi2 somehow strengthens the binding to ubiquitylated conjugates, essentially locking Ddi2 onto its substrates. To test this hypothesis, HEK293T cells treated with the E1 inhibitor TAK-243 to eliminate endogenous ubiquitin conjugates were incubated with or without nelfinavir for 2 h. Cell lysates were added to a glutathione resin containing ubiquitylated GST-E6AP, and the binding of Ddi2 to these Ub conjugates was measured. Inhibiting Ddi2 with nelfinavir increased the amount of Ddi2 bound to the ubiquitylated proteins (Fig. 5*D*). Similar experiments were then carried out with lysates from HCT116 cells to compare the effects of the D252N mutant and WT Ddi2. Nearly twice as much Ddi2 from the D252N cells bound to Ub(n)E6AP (Fig. 5*E*) than WT Ddi2. Thus, nelfinavir or the loss of the protease activity of Ddi2 clearly enhanced the binding of Ddi2 to Ub conjugates.

Surprisingly in these experiments, the levels of another shuttling factor, ubiquilin-1 (UBQLN1), but not of Rad23B, also increased in association with ubiquitylated E6AP in the presence of nelfinavir, even though UBQLN1, unlike Ddi2, does not have a protease domain. Furthermore, in the Ddi2 D252N mutant cells, UBQLN1 levels recovered on Ub(n)E6AP also tended to be higher than in WT cells. Because the loss of the proteolytic activity of Ddi2 or treatment with nelfinavir enhanced the binding of UBQLN1 as well as Ddi2, it seems possible that UBQLN1 and Ddi2 bind to the same Ub conjugates.

Since inactivating Ddi2 increased its binding to Ub conjugates (and possibly UBQLN1), we tested whether inhibiting Ddi2 also increases its association with proteasomes. We treated HEK293T-Rpn11-HBTH cells with 20 μM nelfinavir for 2 h. (The Rpn11 proteasome subunit in these cells is derivatized to allow rapid 26S isolation). Lysates of these cells were added to a streptavidin resin to capture 26S proteasomes. When cells were treated with nelfinavir, significantly less Ddi2 (and less UBQLN1) were bound to proteasomes than in untreated controls (Fig. 5*F*). Thus, although inhibiting Ddi2 increases its binding to Ub conjugates, this drug disrupts the binding of Ddi2 to 26S proteasomes.

### Loss of Ddi2 activity decreases overall rates of protein degradation

**Figure 6:**
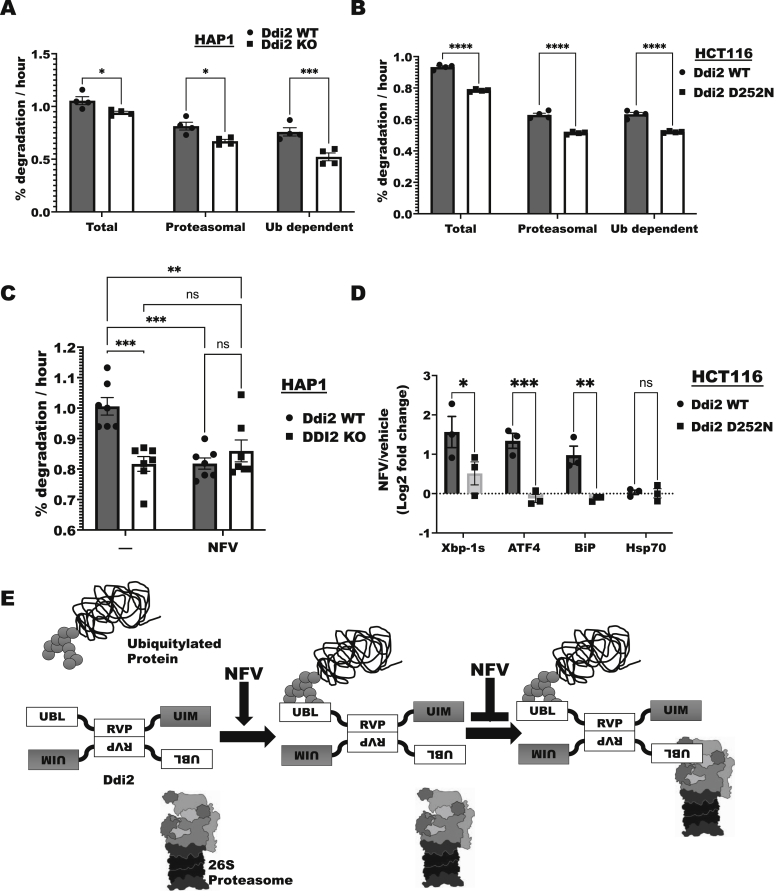
**Loss of Ddi2 or its protease activity causes a small decrease in overall total protein degradation in cells by the Ub–proteasome pathway and an activation of the unfolded protein response.***A*, deletion of Ddi2 decreases total, proteasomal, and Ub-dependent degradation of cell proteins. WT and Ddi2 KO HAP1 cells were incubated with ^3^H-phenylalanine for 20 h to label the bulk of cell proteins (long-lived fraction) and were washed twice to remove labeled precursor and resuspended in chase medium containing a large excess of nonradioactive phenylalanine. Degradation of radiolabeled proteins was then measured by the appearance of ^3^H-phenylalanine (*i.e.*, trichloracetic acid-soluble radioactivity) in the medium during the 6 h chase period. These rates of degradation were linear for 6 h and highly reproducible (45). Proteasomal degradation was determined as the amount of degradation that was inhibited by 10 μM bortezomib during the chase period (44). Ub-dependent degradation is the degradation that was inhibited by 10 μM TAK-243 (n = 4; mean; ∗*p* < 0.05, ∗∗∗*p* < 0.005). *B*, loss of the proteolytic activity of Ddi2 decreased total, proteasomal, and Ub-dependent degradation of cell proteins. Proteins were radiolabeled in HCT116 Ddi2 WT D252V, and their degradation as in (*A*) (n = 4; mean; ∗∗∗∗*p* < 0.001). *C*, the protease inhibitor nelfinavir (NFV) inhibits protein degradation in Ddi2 WT cells but not in DDI2 knockout HAP1 cells. Degradation was measured as in (*A*) after cells were treated with 20 μM NFV or an equivalent volume of methanol (vehicle) during the chase period (n = 8; mean; ∗∗*p* < 0.01, ∗∗∗*p* < 0.005). *D*, NFV caused induction of the unfolded protein response in the ER through an effect on the proteolytic activity of Ddi2, HCT116 Ddi2 WT, and D252N knock-in cells were treated with 10 μM NFV or methanol (vehicle) for 9 h. Data are expressed as the Log_2_ fold change in expression of NFV over control (n = 3; mean; ∗*p* < 0.05, ∗∗*p* < 0.01, and ∗∗∗*p* < 0.005). *E*, a model summarizing the binding of NFV increasing the binding of Ddi2 to ubiquitylated proteins, whereas decreasing the binding of Ddi2 to 26S proteasomes. Ddi2 binds to ubiquitylated proteins, which can contain branched Ub linkages, through its UBL domain. This UBL domain is also used to bind 26S proteasomes, though we suspect *via* different ends of the dimerized molecule. The retroviral protease (RVP) domain mediates dimerization. The UIM domain’s role remains unclear. When the Ddi2 protease is inhibited, the binding to ubiquitylated proteins is favored, whereas the binding to 26S proteasomes is stymied. ER, endoplasmic reticulum; Ub, ubiquitin; UBL, Ub-like domain; UIM, Ub-interacting motif.

Because Ddi2 appears to function as a shuttling factor, and the loss of the proteolytic activity of Ddi2 causes a buildup of large Ub conjugates, we tested if deletion or mutation of Ddi2 affected protein degradation in cells. To label the bulk of cell proteins, HAP1 cells were incubated with ^3^H-phenylalanine for 20 h, and then the cells were washed twice and resuspended in a chase medium containing very high amounts of nonradioactive phenylalanine (44). This approach allows highly precise measurements of overall degradation rates in cells (45). The deletion of Ddi2 resulted in a small, but highly reproducible, decrease (about 10%, *p* < 0.05) in rates of degradation of these long-lived proteins (Fig. 6*A*), which represent the great majority of cellular proteins (46). We then studied if Ddi2 promotes degradation through the Ub–proteasome pathway by determining whether this process was sensitive to bortezomib or TAK-243. As previously observed (47), 80% of total degradation in control HAP1 cells required the proteasome and ubiquitylation (Fig. 6*A*). Deletion of Ddi2 resulted in a 10 to 25% decrease in Ub–proteasome–dependent degradation of long-lived proteins (Fig. 6*A*), which can account for the reduction in overall cell proteolysis.

To determine if deletion of Ddi2 affected overall degradation through loss of its proteolytic activity, we examined protein degradation by the Ub–proteasome pathway in HCT116 with WT Ddi2 or the D252N mutation. This possibility seems likely because mutation of the Ddi2 active site also caused an accumulation of large Ub conjugates and greater association of Ddi2 with ubiquitylated proteins, while also decreasing the binding to 26S proteasomes. As found with the Ddi2 deletion, loss of the endoproteolytic activity of Ddi2 led to about a 15% reduction (*p* < 0.05) in total protein breakdown (Fig. 6*B*). Moreover, the Ddi2 D252N mutation decreased similarly proteasome-dependent and Ub-dependent proteolysis (Fig. 6*B*). Thus, the protease activity of Ddi2 seems to promote protein degradation through the Ub–proteasome pathway. These findings predict that the retroviral protease nelfinavir should decrease overall protein degradation rates. Accordingly, addition of nelfinavir to prelabeled cells caused about a 15% decrease in overall degradation rates in WT HAP1 cells (Fig. 6*C*), which was similar to the decrease observed with the deletion of Ddi2 in HAP1 cells. Importantly, nelfinavir did not cause a significant reduction in degradation rates in Ddi2 knockout cells (Fig. 6*C*). However, these findings do not distinguish whether Ddi2 is of major importance in the degradation of a small fraction of normally long-lived cell proteins, or if it contributes to a small extent to the degradation of many cell proteins by the Ub–proteasome pathway.

### Nelfinavir induces the UPR through Ddi2 inhibition

Nelfinavir has been reported to induce the ER UPR (37, 48, 49). Because Ddi2 was required for nelfinavir to decrease protein degradation rates (Fig. 6*C*), it seemed likely that Ddi2 would be involved in nelfinavir’s induction of the UPR. Therefore, we measured in HCT116 cells the induction of the UPR signaling proteins, spliced Xbp-1 and activating transcription factor 4, by immunoblotting after treatment of WT and Ddi2 D252N cells with or without nelfinavir for 9 h. In the WT cells, induction of these signaling proteins more than doubled with nelfinavir (Fig. 6*D*), but in the Ddi2 D252N mutant, there was little or no induction of these genes. The ER-resident chaperone binding immunoglobulin (Ig) protein (BiP) is also induced as part of the UPR. We found that its induction by nelfinavir also required the Ddi2 active site (Fig. 6*D*). In contrast, nelfinavir did not induce the cytosolic paralog of BiP, Hsp70. Thus, nelfinavir does not induce the heat shock response, which reflects the accumulation of misfolded proteins in the cytosol, but inhibiting the proteolytic activity of Ddi2 causes ER stress and may reduce degradation of proteins through the ERAD pathway.

## Discussion

These studies have established that Ddi2, despite its unusual features (*i.e.*, the presence of a protease domain and the absence of a typical Ub-binding domain), can function as a shuttling factor. Moreover, the loss of Ddi2 function decreases overall protein degradation rates and induces ER stress. Perhaps the most unusual consequence of the loss of Ddi2 function is the accumulation in the cells of very slowly migrating Ub conjugates. Such apparently “large” ubiquitinated proteins accumulated not only in Ddi2-knockout strains (29) (Fig. 1*A*) but also in cells with a mutation in the proteolytic site of Ddi2 (Fig. 4, *B* and *C*). Similar slowly migrating conjugates were observed by Leto *et al.* (36) in their study of ERAD. They found that ERAD substrates migrated unusually slowly after inhibition of the AAA+ unfoldase p97/VCP but not when proteasomes were inhibited. These slowly migrating Ub chains contained K11–K48 branched ubiquitin chains. Likewise, we found that deleting Ddi2 caused an accumulation of slowly migrating ubiquitylated proteins that did not occur upon inhibition of proteasomes alone (Fig. 1*A*). Moreover, these ubiquitylated proteins were shown by immunoblot to contain K11–K48 branched Ub chains (Fig. 1*C*). When Ddi2 was deleted, the buildup of slowly migrating ubiquitylated proteins increased further upon proteasome inhibition (Fig. 1*A*). Therefore, in the absence of a functional Ddi2, such Ub conjugates may not be delivered efficiently to proteasomes but are probably still being slowly degraded by proteasomes. Ddi2 also was found to bind selectively to such slowly migrating Ub conjugates (Fig. 1, *B* and *D*). So, their accumulation upon deletion or mutation of Ddi2 strongly suggests that these slowly migrating ubiquitylated proteins are substrates of its proteolytic activity.

These observations also suggest that Ddi2 functions as a shuttling factor in ERAD. In their comprehensive screen for genes regulating the degradation of proteins by ERAD, Leto *et al.* (36) knocked out each of the mammalian homologs of the yeast UBL–UBA shuttling factors. Although it was not noted in their text, data in the supplement to their article clearly show that knockout of Ddi2, but not of other shuttling factors, increased the stability of the four ERAD substrates tested (see Table 1 based on their data). Therefore, Ddi2 is likely to serve an important, but previously unrecognized, role in the degradation of proteins derived from the ER. Such a conclusion is also supported by our observation that Ddi2 inhibition by nelfinavir induces the ER stress response (Fig. 6*D*). A role for Ddi2 in ERAD seems quite analogous to its role in the proteolytic activation of the ER-associated transcription factor Nrf1 (25) where Ddi2 functions after and before proteasomes. However, in a yeast screen for genes affecting ERAD (12), Ddi1 (unlike Rad23 and Dsk2) was not identified. It is not clear if this apparent lack of an effect of Ddi1 knockout is due to redundancy of shuttling factors in yeast or to a fundamental difference in the function of yeast Ddi1 and mammalian Ddi2.

**Table 1:** Stabilizing effects of shuttling factors on ERAD substrates measured by Leto *et al.* (36)

Gene	A1AT^NHK^-GFP		GFP-RTA^E177Q^		INSIG1-GFP		GFP^u^	
	Effect size	*p*	Effect size	*p*	Effect size	*p*	Effect size	*p*
Ddi1	−6.0 to −0.2	0.569	−1.8 to 0.8	0.661	−2.5 to 1.4	0.903	−0.6 to 2	0.742
Ddi2	0.9–1.9	0.0213	**1.6–3.0**	**0.000002**	**2.3–3.6**	**0.000002**	**2.3–3.4**	**0.000002**
Rad23A	−0.7 to 1.8	0.863	0.5–1.4	0.0239	0.2–3.7	0.444	−3.6 to 1.4	0.941
Rad23B	1.2–5.4	0.0316	0.5–1.6	0.0273	−3.0 to 4.0	0.720	−2.7 to 1.9	0.754
UBQLN1	−1.6 to 1.1	0.831	−3.5 to 1.4	0.931	−1.1 to 1.8	0.922	−3.1 to 1.4	0.730
UBQLN2	−1.8 to 1.2	0.984	−2.0 to 0.7	0.889	−1.6 to 1.3	0.917	−2.6 to 1.3	0.824
UBQLN3	−2.5 to 1.7	0.846	−2.0 to 0.5	0.699	0.1 to 2.5	0.417	−2.5 to 2.0	0.761
UBQLN4	0.5–1.8	0.171	−2.8 to 1.2	0.724	0.7–2.0	0.0315	0.4–3.4	0.183
UBL7	0.3–5.0	0.531	−2.8 to 1.1	0.931	0.4–1.5	0.174	−4.5 to 1.5	0.602

Bold highlights effects that surpassed the false discovery rate correction for significance.

Although Ddi2 has several features that had suggested that it could not function as a shuttling factor, our data clearly support such a role. Surprisingly, Ddi2 binds to ubiquitylated proteins through its UBL domain, even though it also uses its UBL domain to bind to 26S proteasomes (Fig. 1*E*). This finding is consistent with the proposal that in yeast, Ddi1 may also bind Ub chains through its UBL domain (18). Presumably Ddi2 can use its UBL domain to simultaneously bind both 26S proteasomes and Ub conjugates because Ddi2 functions as an antiparallel dimer (19) and thus would have two UBL domains per functional unit (Fig. 6*E*). Probably the strongest evidence for its ability to act as a shuttling factor is our observation that using purified proteins, Ddi2 addition can promote the binding of Ub conjugates to proteasomes (Fig. 3*A*), and in cell extracts, its association with 26S particles is dependent on a supply of Ub conjugates (Fig. 2, *B* and *C*), as we had found with Rad23B (21).

Cells lacking Ddi2 or with an inhibited Ddi2 protease site degrade proteins more slowly by the Ub–proteasome pathway (Fig. 6, *A*–*C*), as shown by pulse-chase isotopic assays that monitor the hydrolysis of the bulk of cell proteins. The degradation of these long-lived cell components can be measured very precisely (45). The small (∼15–20%) but highly reproducible defect in their degradation upon the loss of Ddi2 activity is presumably a consequence of its role as a shuttling factor that delivers Ub conjugates to the proteasome. The reduction in overall proteolysis upon loss of Ddi2 function may indicate either a modest decrease in the degradation of most cell proteins, or perhaps a major defect in the breakdown of a specific class of cell proteins, most likely those bearing slowly migrating branched ubiquitin chains (see aforementioned). However, the small reduction in total degradative rates probably underestimates the true importance of Ddi2 because the several shuttling factors in mammalian cells probably have overlapping activities, as has been clearly shown in yeast for Rad23 and Ddi1 (50, 51). Accordingly, in mammalian cells, it was necessary to knockdown both Ddi1 and Ddi2 to observe defects in DNA repair (52). Moreover, we found that Rad23B, like Ddi2 binds, preferentially to the slowly migrating branched conjugates. In fact, Ddi2 shares two other interesting properties with Rad23B: it can protect ubiquitylated chains from cytosolic DUbs (Fig. 5, *B* and *C*), and Ddi2 only binds to 26S proteasomes in cell lysates if Ub conjugates are present (Figs. 2, *B*–*D* and 3*B*).

Although these observations clearly demonstrate that the proteolytic activity of Ddi2 is physiologically important, the specific functions of the protease activity linked to a shuttling factor are still enigmatic. Perhaps the Ddi2 protease functions as a safety mechanism, cutting ubiquitylated substrates that 26S proteasomes fail to process efficiently. Accordingly, the cleavage by Ddi2 of membrane-bound Nrf1 to release its active form is clearly evident only when proteasomes are inhibited (25). Another possible function of the protease might be to allosterically regulate how Ddi2 binds ubiquitylated proteins and hands them off to proteasomes. Surprisingly, the protease domain of Ddi2 affects the binding of Ddi2 to ubiquitylated conjugates and to proteasomes in opposite ways. Inhibition or mutation of the Ddi2 protease site increased the association of Ddi2 with ubiquitylated proteins (and to UBQLN1) but decreased Ddi2 binding to 26S proteasomes (Fig. 6*E*). Thus, depending on the engagement of the protease domain with a ubiquitylated protein, Ddi2 shifts from a structure that has high Ub and low proteasome affinity to a conformation with lower Ub and higher proteasome affinity. In other words, the cleavage of a ubiquitinated protein by Ddi2 may increase its ability to deliver the Ub conjugate to the proteasome. Although the proteolytic domain of retroviral proteases is essential for their dimerization, inhibitors of the HIV protease do not disrupt Ddi2 dimerization (51). Also, the Ddi2 protease mutant does not appear to affect the shuttling of Ub conjugates by preventing dimerization. In fact, expression of the Ddi2 DV/QL mutant resulted in a dominant negative inhibition of WT Ddi2 with which it formed a complex (data not shown).

Combinations of nelfinavir with proteasome inhibitors have been shown to have enhanced cytotoxic effects (31, 32). Consequently, clinical trials are exploring the potential for nelfinavir, in combination with the proteasome inhibitor, bortezomib, to enhance the treatment of multiple myeloma (53, 54). Although nelfinavir was reported to inhibit directly the 20S proteasome’s active sites (37, 55, 56), no such effect is evident with nelfinavir concentrations studied here (data not shown) and cannot explain this additivity. Cells adapt to proteasome inhibition by Nrf1-dependent activation of the expression of genes for new proteasomes, as well as p97 and p62, and by blocking this adaptive response, nelfinavir may increase the toxicity of bortezomib (25, 31, 32, 57). However, as shown here, because nelfinavir by inhibiting Ddi2 decreases total cellular protein breakdown (Fig. 6*C*) and causes the induction of ER stress responses (Fig. 6*D*), nelfinavir has similar cellular effects as treatments with proteasome inhibitors. Thus, the additive effects of nelfinavir and proteasome inhibitors in mediating the killing of cancer cells likely involve several mechanisms, which are dependent on Ddi2.

## Experimental procedures

### Cell culture

HAP1 cells and HAP1 Ddi2 knockout cell lines were obtained from Horizon (HZGHC000182c006) and were cultured in Iscove's modified Dulbecco's medium (Gibco) with 9% fetal bovine serum (FBS). HAP1 lines stably expressing shRNA to Nrf1 were generated by lentiviral particles expressing shRNAs for Nrf1 (Sigma; catalog no.: TRCN0000280355). As controls, cells were infected with lentiviral particles expressing copGFP (Santa Cruz; catalog no.: sc-108084). Lentivirus infection was set up in 24-well format with 2.5 × 10^4^ cells per well. About 50,000 lentiviral particles were mixed in complete medium in a total volume of 250 μl, and polybrene (Santa Cruz; catalog no.: sc-134220) was added to a final concentration of 8 μg/ml. The mixture was applied to each well for 6 h and then replaced with 350 μl fresh complete medium. One day after infection, stable clones were selected for in the presence of 1 μg/ml puromycin.

HEK293A, HEK293-Dss1-FLAG cells (58), and HEK293F-Rpn11-HBTH cells (59) were cultured in Dulbecco’s modified Eagle’s medium with 4.5 g/l glucose, l-glutamine, and sodium pyruvate with 9% FBS.

HCT116 WT and Ddi2 D252N cells (24) were cultured in McCoy’s 5A modified medium, with l-glutamine and sodium bicarbonate with 9% FBS. All cells were cultured in humidified, 37 °C, 5% CO_2_ incubators.

### Plasmids and transfection

The pReceiver-M02-Ddi2 was purchased from GeneCopoeia (EX-Z2704-M02-B). To express hemagglutinin (HA)-Ddi2, Ddi2 from pReceiver-MO2-Ddi2 was excised by digestion with BstBI (New England Biolabs; catalog no.: R0519S) and NotI (NEB; catalog no.: R0189S) and cloned into the BstBI/NotI sites of the pReceiver-M06 plasmid (GeneCopoeia). To construct vectors to express other tagged and/or truncated Ddi2, PCR using Phusion High-fidelity PCR Master Mix with GC buffer (NEEB; catalog no.: M0532S) was used to amplify Ddi2 from the pReceiver-MO2-Ddi2 plasmid. To express FLAG-Ddi2, the 5′ primer was GGCGAATTCATGC TGCTCACCGTGTACTGT and the 3′ primer was TTTGGG CCCCTATGGCTTCTGACGCTCT. Amplified full-length Ddi2 DNA was digested with EcoRI (NEB; catalog no.: R3101S) and ApaI (NEB; catalog no.: R0114S) and cloned into EcoRI/ApI sites of pCMVTAG2B (Stratagene; catalog no.: 211172). Since the starting codon of Ddi2 was not disrupted when constructing vectors to express HA-Ddi2 or FLAG-Ddi2, these vectors also induce some untagged Ddi2. To express HA-Ddi2ΔUBL, the 5′ primer was ACCTTCGAACAGACCC TCGACCTCCAGTG and the 3′ primer was AAAGCGGCC GCACTCGAGCTATGGCT. The amplified Ddi2ΔUBL DNA was cloned into the BstBI/NotI sites of pReceiver-M06. To express FLAG-Ddi2ΔUBL, the 5′ primer was GGCGAATTCATGCTGCTCACCGTGTACTGT and the 3′ primer was TTTGGGCCCCCTATGGCTTCTGACGCTCT. The amplified Ddi2ΔUBL complementary DNA was cloned into the EcorRI/ApaI sites of pCMVTAG2B. To express the protease-inactive Ddi2 DV/QL, the mutation was introduced by the QuikChange II Site-Directed Mutagenesis Kit (Agilent; catalog no.: 200523), using the GCTCATGATAGTCATCAGGGCC CCTGAGACAACAAAGGCTTTCAC, GTGAAAGCCTTTG TTGTCTCAGGGGCCTGATGACTATCATGAGC primer pairs, into pReceiver-M02-Ddi2, pReceiver-M06-Ddi2, and pCMVTAG2B-Ddi2 plasmids. The pReiver-M02-Ddi2 DV/QL plasmid was further used as PCR template to clone Ddi2 ΔUBL DV/QL. To construct FLAG-Ddi2 ΔUIM, the 5′ primer is GGCGAATTCATGCTGCTCACCGTGTACTGT and the 3′ primer is TTTGGGCCCTCACTCTGGTAGCTCTCCCTC; and to construct FLAG-Ddi2 ΔΔ, the 5′ primer is AAAGAAT TCGACCCTCGACCTCCAGTGCAG and the 3′ primer is TTTGGGCCCTCACTCTGGTAGCTCTCCCTC. Amplified fragments were cloned into EcoRI/ApaI sites of pCMVTAG2B.

HEK293A cells were cultured in 6-well plates to 80% confluency. Transfection mixture containing 10 μg DNA and 5 μl Lipofectamine 2000 (Thermo Fisher Scientific; catalog no.: 11668-019) was prepared in 500 μl Opti-MEM I Reduced-Serum Medium (Thermo Fisher Scientific; catalog no.: 51985-034) and allowed to mix at room temperature for 20 min and then added to each well containing 2.5 ml complete medium. After 8 h, the transfection mixture was replaced with fresh medium. HAP1 and HCT116 cells were cultured in 12-well plates until 50% confluence. Transfection mixtures containing 2 μg DNA and 6 μl FugeneHD (Promega; catalog no.: E2311) were prepared in 100 μl Opti-MEM medium and incubated at room temperature for 15 min before addition to each well containing 1 ml complete medium. Cells were cultured for another 48 to 72 h before assays. Mock transfection was performed with a plasmid to express GFP (pCMV-GFP).

### Small molecules and treatment

Stock solutions were prepared for the following inhibitors: bortezomib (LC Laboratories; catalog no.: B-1408; 100 mM in dimethyl sulfoxide [DMSO]), NMS-873 (Sigma; catalog no.: SML1128; 10 mM in DMSO), pifithrin μ (Selleckchem; catalog no.: S2930; 20 mM in DMSO), TAK-243 (Active Biochem; catalog no.: A1384; 10 mM in DMSO), nelfinavir (Cayman Chemical; catalog no.: 15144; 20 mM in methanol), and Ub-aldehyde (Boston Biochem; catalog no.: U201; 1600 μM in 50 mM HCl).

### FLAG- Ddi2 pulldowns

Cell lysates were prepared in 25 mM Hepes (pH 7.4), 1 mM ATP, 10 mM MgCl_2_, 0.2% NP-40, and 10% glycerol with sonication (1 × 10 s +3 × 5 s at 12 μm amplitude) and cleared by centrifugation for 10 min at 10,000*g* at 4 °C. Cleared input lysate at a protein concentration of 1 to 2 μg/μl was subject to immunoprecipitation with 10% volume of anti-FLAG (M2) affinity gel (Sigma; catalog no.: A2220) by rocking for 5 h at 4 °C. Unbound proteins were washed away by washing the resin with 5 × 10 column volume of Hepes lysis buffer. Proteins bound to the affinity gel were eluted at 95 °C in Laemmli buffer.

### Purified proteins

#### His-Ddi2

His Ddi2 plasmid (pET16b), a gift from Klara Saskova (Charles University), was used for expression in BL21 RIL (DE3) Codon Plus *Escherichia coli* cells (Agilent). Protein expression was induced for 16 h at 16 °C with 0.5 mM IPTG when the cultures reached absorbance of 0.6 to 0.8 at 600 nm. Bacteria were lysed in 50 mM NaH_2_PO_4_, 300 mM NaCl, and 10 mM imidazole (pH 8), by passing through an emulsiflex homogenizer (Avastin) at 10,000 psi. Lysates were clarified by centrifugation (100,000*g*, 30 min) and bound to Ni–nitrilotriacetic acid (NTA) agarose (Gold Biotechnology; catalog no.: H-350) at 4 °C for 1 h. The resin was washed three times with 10 column volumes of 50 mM NaH_2_PO_4_, 300 mM NaCl, and 20 mM imidazole (pH 8) before elution with one column volume of 50 mM NaH_2_PO_4_, 300 mM NaCl, and 250 mM imidazole (pH 8). Ddi2-positive fractions were pooled and concentrated with a spin filter (Amicon Ultra 10,000 molecular weight cutoff) before injection into a Superdex 2000 16/60 column (GE Healthcare). Ddi2 was eluted with 25 mM Hepes–KOH (pH 8), 40 mM potassium acetate, 10% (v/v) glycerol, and 1 mM DTT. Ddi2 was collected at a molecular weight consistent with dimerization.

#### 26S Proteasomes

26S Proteasomes from mouse embryonic fibroblasts were purified by UBL affinity method as described (40). Proteasomes from HEK293F cells stably overexpressing the FLAG-tagged DSS1 (58) were purified by FLAG resin (Anti-FLAG M2 affinity Gel; Sigma) as described (60).

##### E6AP and Nedd4

GST-E6AP and GST-Nedd4 were expressed in BL21 *E. coli*, purified, and autoubiquitylated as previously described (61). NeD-sfGFP was expressed, purified, ubiquitylated, and labeled as previously described (62). NeD-sfGFP was expressed in BL21 DE3 RIPL *E. coli* (Agilent) in Terrific Broth medium by induction with 0.5 M IPTG for 16 h at 16 °C once the culture reached an absorbance of 1 at 600 nm. Cells were harvested by centrifugation and lysed in 50 mM Tris–HCl (pH 8), 200 mM NaCl, 30 mM imidazole, 1 mM PMSF, and 5 mg/l DNase I by sonication. Lysates were clarified by centrifugation (100,000*g*, 30 min, 4 °C). Supernatants were incubated with Ni–NTA agarose resin at 4 °C for 90 min. The resin was washed three times with 20 column volumes of 50 mM Tris–HCl (pH 8), 200 mM NaCl, and 30 mM imidazole before proteins were eluted twice with 50 mM Tris–HCl (pH 8), 100 mM NaCl, and 400 mM imidazole. Once the two eluted fractions were combined, they were supplemented with 1 mM Tris(2-carboxyetheyl)phosphine (TCEP). The His14-SUMO tag was removed by incubation with 2 μM Ulp1 (SUMO protease) at 4 °C for 2 h before dialysis in 50 mM Hepes (pH 7) and 50 mM NaCl. NeD-sfGFP was then loaded onto a MonoQ 10/100 GL column and eluted with a linear gradient from 50 mM NaCl, 50 mM Hepes (pH 7) to 500 mM NaCl, 50 mM Hepes (pH 7) over 10 column volumes. Peak fractions were pooled and concentrated to at least 2 mg/ml and stored at −80 °C.

Prior to labeling, the buffer for NeD-sfGFP was exchanged to 50 mM Hepes (pH 7) and 150 mM NaCl using a PD-10 desalting column. Proteins were then labeled with 5:1 M excess of DyLight 800 maleimide for 1 h at room temperature. The reaction was quenched by addition of 1 mM DTT and cleaned up with Pierce Dye Removal Columns (Thermo Fisher Scientific).

About 500 nM NeD-sfGFP was ubiquitylated with 100 μM *S. cerevisiae* ubiquitin, 100 nM Uba1, 12 μM Ubc2, and 300 nM Ubr1 (gifts from Tom Rapoport) in 50 mM Tris–HCl (pH 8), 150 mM NaCl, 10 mM MgCl_2_, 5 mM ATP, and 1 mM DTT at 30 °C for 45 min. NeD-sfGFP was then bound to Streptavidin beads (Thermo Fisher Scientific) and incubated at 4 °C for 1 h. The resin was washed twice with 10 column volumes of 50 mM Tris–HCl (pH 8), 150 mM NaCl, 10 mM MgCl_2_, 5 mM ATP, and 1 mM DTT. NeD-sfGFP was eluted twice with two column volumes of 40 mM biotin in 50 mM Tris–HCl (pH 8), 150 mM NaCl, and 1 mM DTT. NeD-sfGFP was then treated with 2 μM 3C protease on ice for 2 h and then gel filtered through a Superdex 200 10/300 column using 50 mM Hepes (pH 7.4), 150 mM NaCl, and 0.5 mM TCEP. Ubiquitylated NeD-sfGFP fractions were pooled, and its concentration was determined by fluorescence relative to nonubiquitylated NeD-sfGFP.

### Binding assays

#### Measurement of His-Ddi2 binding to proteasomes

Ddi2 and 5 nM purified proteasomes were combined in 25 mM Hepes–KOH (pH 8), 125 mM potassium acetate, 1 mM DTT, 2.5 mM MgCl_2_, 0.1 mg/ml bovine serum albumin (BSA), 0.05% Triton X-100, 1 mM ATP, and a constant volume of Ni–NTA agarose (Gold Biotechnology; catalog no.: H-350) at 4 °C for 30 min. Resin was separated from the unbound fraction by centrifugation (100*g*, 2 min). The column was washed three times with 10 column volumes of 50 mM Tris (pH 7.5), 10 mM MgCl_2_, 40 mM KCl, 1 mM ATP, and 1 mM DTT. Proteasome activity on the resin was measured in 50 mM Tris (pH 7.5), 10 mM MgCl_2_, 40 mM KCl, 1 mM ATP, 1 mM DTT, and 20 μM Suc-Leu-Leu-Val-Tyr-amc at λ_excitation_ 380 nm, λ_emission_ 460 nm, and 37 °C for 30 min. Captured proteasome activity is reported directly as arbitrary fluorescence units.

#### Measurement of endogenous Ddi2 binding to proteasomes in cell lysates

HEK293F cells stably overexpressing FLAG-tagged DSS1 were pretreated with 1 μm bortezomib or 5 μM TAK-243 before cell lysis. Proteasomes were prepared from cell extracts after 100,000*g* spin for 30 min. When indicated, 100 nM Ub_n_-GST-E6AP or Ub_n_-GST-Nedd4 was added to the cell extracts during proteasome pulldown at 4 °C. To measure protein dissociation from the proteasomes, in buffer, resin-bound proteasomes were incubated at 37 °C in wash buffer for 0 to 20 min. After four washes, proteasomes were eluted, and proteins including Ddi2 on purified proteasomes were analyzed by immunoblot.

### Far Western blotting

Far Western blotting was adapted from the protocol described by Wu *et al.* (63). Cells were lysed in 50 mM Tris–HCl (pH 7.4), 150 mM NaCl, 1 mM NaF, 1 mM EDTA, 1 mM Na_3_VO_4_, 1 mM DTT, 1% Triton X-100 with Roche cOmplete Protease Inhibitor. About 60 μg of protein lysate were run on 4 to 12% Bis–Tris plus mini gel and then transferred to 0.45 μm PVDF low-fluorescence membranes. Proteins on the membrane were denatured and allowed to refold by washing in 100 mM NaCl, 20 mM Tris–HCl (pH 7.6), 0.5 mM EDTA, 10% glycerol, 0.1% Tween-20, 2% skim milk, and 1 M DTT with progressively decreasing concentrations of guanidine-HCl (6 M, 3 M, 0.1 M, and no guanidine-HCl). After blocking the membrane in 5% (w/v) milk–Tris-buffered saline with Tween-20, 1 μg/ml Ddi2 or Rad23B in 100 mM NaCl, 20 mM Tris–HCl (pH 7.6), 0.5 mM EDTA, 10% glycerol, 0.1% Tween-20, 2% skim milk, and 1 M DTT was incubated with the membrane at 4 °C, overnight. After washing with Tris-buffered saline with Tween-20, primary antibodies to Ddi2, Rad23B, or ubiquitin (P4D1) were used with an overnight incubation at 4 °C followed by IRDye800-labeled secondary antibodies.

### Initial binding assay

Measurement of the binding of 26S proteasomes to ubiquitylated E6AP was performed as described (61). To 100 nM ubiquitylated GST-E6AP, 500 nM Ddi2 or Rad23B and 10 nM 26S proteasomes were added in 25 mM Hepes–KOH (pH 8), 125 mM potassium acetate, 1 mM DTT, 2.5 mM MgCl_2_, 0.1 g/l BSA, 0.05% Triton X-100, and 1 mM ATP. After binding at 4 °C for 30 min, the resin was washed first in binding buffer and with proteasome assay buffer (50 mM Tris–HCl [pH 7.6], 100 mM KCl, 1 mM ATP, 5 mM MgCl_2_, and 0.025 g/l BSA) and then assayed in that buffer in 10 μM suc-Leu-Leu-Val-Tyr-amc.

### Ddi2 proteolytic assays

About 150 nM labeled Ub(n)NeD-sfGFP was incubated with 150 nM Ddi2 in 50 mM Hepes (pH 7.4), 150 mM NaCl, 0.5 mM TCEP, and 0.01 mg/ml BSA and incubated 37 °C. At the indicated time points, the reaction was stopped with 1× Laemmli loading buffer and incubated at 70 °C for 5 min. The samples were run on Novex 4 to 20% Tris–glycine Mini Gels (Thermo Fisher Scientific) in Tris–glycine SDS running buffer. The gels were scanned with an Odyssey CLx imager.

### Measurement of cellular protein degradation

Cells were labeled for 20 h with phenylalanine (Phe l-[3, 4, 5 ^3^H], American Radiolabeled Chemicals; catalog no.: ART0614, 1 mCi/ml in 0.01 M HCl, final 5 μCi/ml) and then chased for 2 h with complete media containing 2 mM cold Phe (Sigma; catalog no.: P5482). Cells were then incubated in media containing both 2 mM cold Phe and inhibitors of proteasome (bortezomib) or ubiquitylation (TAK-243). Released ^3^H-Phe was precipitated by 0.625 M trichloroacetic acid (TCA) (VWR; catalog no.: BDH3372-2). Media were collected at 0, 1, 2, 3, and 4 h during the incubation. About 200 μl of the supernatant after TCA precipitation was mixed with 3 ml Bio-Safe II Complete Counting Cocktail Scintillation Fluid (RPI; catalog no.: 111195-CS), and the TCA-soluble count was measured. To calculate percent protein degradation, cells were lysed after the last time point with 0.2 M NaOH, and 100 μl lysate was mixed with 3 ml scintillation fluid to measure total radioactivity incorporated. Protein degradation rates were determined by plotting percent protein degradation over time. Four independent samples were used to determine the protein degradation rates. To determine the fraction of protein degradation dependent on proteasome or ubiquitylation, 10 μM bortezomib was added after the chase to inhibit the proteasome, or 10 μM TAK-243 was added to inhibit the Ub-activating enzyme.

### Immunoblot assays

Cells were generally lysed for 30 min in ice-cold 50 mM Tris–Cl (pH 7.4), 150 mM NaCl, 1 mM NaF, 1 mM EDTA, 1 mM Na_3_VO_4_, 1 mM DTT, 1% Triton X-100, and Roche Protease Inhibitor Cocktail Tablet. After centrifugation (10,000*g* for 10 min), the supernatant was used as cleared lysate for Western blotting. For detection of branched Ub chains, we followed the method by Newton *et al.* (64) for detecting Ub conjugates. Cells were lysed in 8 M urea, 20 mM Tris–HCl (pH 7.5), 135 mM NaCl, 1% Triton X-100, 10% glycerol, 1.5 mM MgCl_2_, 5 mM EDTA, 10 μl/ml 100× Halt Protease and phosphatase inhibitors (Pierce), and 2 mM *N*-ethylmaleimide. After lysis, lysates were diluted to 4 M urea and the lysate was cleared as afrementioned. Antibodies for immunoblots and their dilutions include total Ub (mouse IgG_1_ P4D1; Santa Cruz Biotechnology; catalog no.: sc-8017; 1:200 dilution), K48-linked Ub (rabbit IgG D9D5; Cell Signaling Technologies; catalog no.: 8081; 1:1000 dilution), Ddi2 (rabbit IgG; Bethyl; catalog no.: A304-629A; 1:5000 dilution), Nrf1 (rabbit IgG D5B10; Cell Signaling Technologies; catalog no.: 8052; 1:1000 dilution), GAPDH (mouse IgM GAPDH-71.1; Sigma; catalog no.: G8795; 1:40,000 dilution), FLAG (mouse IgG1 M2; Sigma; catalog no.: F1804; 1:10,000 dilution), actin (mouse IgG1 AC-15; Sigma; catalog no.: A3854; 1:25,000 dilution), K11–K48 branched Ub (Genentech; 1 μg/ml), Rad23B (rabbit IgG; Bethyl; catalog no.: A302-306A; 1:2000 dilution), Rpt6 (mouse IgG2b p45-110; Enzo; catalog no.: PW9265; 1:1000 dilution), Rpt5 (mouse IgG2b TBP1-19; Enzo; catalog no.: PW8770; 1:1000 dilution), β5 (rabbit IgG; Bethyl; A303-847; 1:2000 dilution), GST (goat IgG; Boston Biochem; catalog no.: A-30; 1:1000 dilution), UBQLN1 (rabbit IgG D3T7F; Cell Signaling Technologies; catalog no.: 14526; 1:1000 dilution), Rpn1/PSMD2 (rabbit IgG; Bethyl; catalog no.: A303-854A; 1:5000 dilution), E6AP (mouse IgG1 E6AP-330; Sigma; catalog no.: E8655; 1:1000 dilution), spliced Xbp-1 (mouse IgG1 E8C2Z; Cell Signaling Technologies; catalog no.: 27901; 1:1000 dilution), activating transcription factor 4 (mouse IgG1 E4Q4E; Cell Signaling Technologies; catalog no.: 97038; 1:1000 dilution), BiP (rabbit IgG C50B12; Cell Signaling Technologies; catalog no.: 3177; 1:1000 dilution), and Hsp70 (mouse IgG1 C92F3A-5; Enzo; catalog no.: ADI-SPA-810; 1:1000 dilution).

### Quantification and statistical analysis

Quantification of immunoblots was carried out using ImageStudio Lite Software (LI-COR). Statistical significance was determined either by ANOVA with post hoc *t* tests or unpaired *t* test with Prism software (version 9.1.2; GraphPad Software). Curve fitting for Figures 2*A*, 4, *A*, and *B* used one-site specific binding modeling with the Prism software.

## Data availability

The data presented in this study are available upon request.

## Conflict of interest

The authors declare that they have no conflicts of interest with the contents of this article.
